# Metformin’s disruption of gluconeogenesis in type 2 diabetes impairments of the cori and alanine cycles as hidden drivers of metabolic waste accumulation, NF-κBHIF-1α–mediated low-grade inflammation, and multisystem dysfunction

**DOI:** 10.14341/probl13618

**Published:** 2026-07-22

**Authors:** Maher Akl, Amr Ahmed

**Affiliations:** National Research Lobachevsky State University of Nizhny Novgorod Египет; National Research Lobachevsky State University of Nizhny Novgorod Egypt; The public health department, Riyadh First Health Cluster Саудовская Аравия; The public health department, Riyadh First Health Cluster Saudi Arabia

**Keywords:** Metformin, Gluconeogenesis Inhibition, Metabolic Inflammation, T2DM Multisystem Effects, Cori Cycle Disruption, Alanine Cycle Impairment, Metformin, Gluconeogenesis Inhibition, Metabolic Inflammation, T2DM Multisystem Effects, Cori Cycle Disruption, Alanine Cycle Impairment

## Abstract

Gluconeogenesis, a dual-purpose pathway in type 2 diabetes mellitus (T2DM), not only synthesizes glucose but also clears metabolic waste via the Cori cycle (lactate recycling through LDH, PC, PEPCK) and Alanine cycle (nitrogen disposal via ALT, GDH, urea cycle), preventing acidosis, ROS accumulation, and ammonia toxicity. Metformin, the cornerstone T2DM therapy, inhibits gluconeogenesis by targeting mitochondrial complex I, elevating AMP/ATP ratios, and activating AMPK-PKCι/λ signaling to repress CREB-CRTC2-driven PEPCK/G6Pase expression, disrupting these cycles. This leads to lactate, pyruvate, and ammonia buildup, triggering pro-inflammatory cascades: HIF-1α stabilization induces IL-6/VEGF, ROS from pyruvate excess activates NF-κB for TNF-α, and ammonia primes NLRP3 inflammasome for IL-1β/IL-18 release, fostering chronic inflammation. Multisystem consequences include musculoskeletal fatigue from ATP deficits, cognitive fog via neuroinflammation, atherosclerosis from endothelial dysfunction, hepatic fibrosis from urea cycle stress, and immune inflammaging impairing macrophage function. Clinical evidence reveals short-term anti-inflammatory benefits (reduced IL-6, CRP) via AMPK and microbiota effects, contrasted by long-term risks like lactic acidosis and neurodegeneration in renal-impaired or elderly patients. This review integrates physiological roles, molecular mechanisms, inflammatory pathways, systemic impacts, and clinical findings, highlighting metformin’s dual-edged profile glycemic efficacy versus “inflammatory debt.” Researchers are urged to explore precision interventions, such as antioxidants or biomarker-guided dosing, to optimize metformin’s pleiotropic potential in T2DM and inflammaging-related disorders, redefining therapeutic paradigms.

## 1. Introduction

Gluconeogenesis, the endogenous synthesis of glucose from non-carbohydrate precursors, is conventionally recognized for its role in maintaining euglycemia during fasting or metabolic stress [1]. However, emerging perspectives underscore its function as a sophisticated metabolic scavenger system, integral to the clearance of potentially deleterious byproducts such as lactate, pyruvate, and nitrogenous compounds. This dual-purpose pathway operates through interconnected cycles, notably the Cori cycle and the Alanine cycle, which facilitate the recycling of metabolic intermediates while averting cellular and systemic perturbations [2][3].

In the Cori cycle, lactate generated primarily in skeletal muscle during anaerobic glycolysis via the action of lactate dehydrogenase (LDH) converting pyruvate to lactate is transported to the liver. Here, LDH reverses the reaction, regenerating pyruvate, which enters the gluconeogenic pathway. Pyruvate carboxylase (PC) catalyzes the carboxylation of pyruvate to oxaloacetate (OAA) in the mitochondrial matrix, utilizing biotin as a cofactor and ATP as an energy source [2][4]. Subsequently, OAA is shuttled to the cytosol via the malate-aspartate shuttle, where phosphoenolpyruvate carboxykinase (PEPCK) decarboxylates and phosphorylates it to form phosphoenolpyruvate (PEP). This intermediate progresses through enolase, phosphoglycerate mutase, and other enzymes to yield glucose-6-phosphate, ultimately hydrolyzed by glucose-6-phosphatase (G6Pase) to free glucose [5]. This cycle not only replenishes circulating glucose but also prevents lactic acidosis by efficiently removing excess lactate, thereby maintaining extracellular pH homeostasis and mitigating the activation of acid-sensing ion channels that could exacerbate inflammatory signaling [2][6].

Complementarily, the Alanine cycle addresses nitrogen disposal and carbon skeleton recycling [3]. Alanine, derived from muscle proteolysis or transamination of pyruvate via alanine aminotransferase (ALT), carries amino groups to the liver. Upon arrival, ALT transfers the amino group to α-ketoglutarate, yielding glutamate and regenerating pyruvate for gluconeogenesis [7].

The amino nitrogen integrates into the urea cycle: glutamate dehydrogenase (GDH) liberates ammonia, which enters the ornithine-citrulline-arginine pathway, culminating in urea synthesis and excretion [8]. This process averts ammonia toxicity, which could otherwise impair mitochondrial function through glutamine synthetase overload or induce hyperammonemic encephalopathy-like states [9][10]. Moreover, the shared reliance on mitochondrial pyruvate carrier (MPC2) for pyruvate import underscores the cycles’ interdependence, ensuring redox balance via NADH/NAD+ ratios and preventing reactive oxygen species (ROS) accumulation from uncoupled electron transport [11].

These cycles collectively safeguard against metabolic acidosis, oxidative stress, and nitrogenous waste buildup, which, if unchecked, could propagate low-grade inflammation through mechanisms such as hypoxia-inducible factor-1α (HIF-1α) stabilization under lactate excess or nuclear factor-κB (NF-κB) activation via ROS-mediated IκB kinase phosphorylation [12]. In healthy physiology, gluconeogenesis supports immune surveillance by providing glucose to activated leukocytes during stress, mimicking Warburg effect shifts where aerobic glycolysis favors rapid ATP and biosynthetic intermediates [13]. Additionally, efficient waste clearance preserves hepatic mitochondrial integrity, cardiovascular endothelial quiescence, and neurological synaptic efficacy by limiting pro-inflammatory cytokine cascades [14].

Type 2 diabetes mellitus (T2DM), a global epidemic affecting over 500 million individuals and characterized by insulin resistance and hyperglycemia, necessitates pharmacological interventions to restore glycemic control [15]. Metformin, a biguanide derivative, stands as the first-line therapy, prescribed to millions for its efficacy in suppressing hepatic glucose output. Its primary mechanism involves inhibition of mitochondrial complex I, elevating the AMP/ATP ratio and activating AMP-activated protein kinase (AMPK), which phosphorylates downstream targets like protein kinase C iota/lambda (PKCι/λ) to disrupt cAMP response element-binding protein (CREB)-CREB-binding protein (CBP) interactions, thereby repressing PEPCK and G6Pase transcription [16]. While this attenuates hyperglycemia, the long-term ramifications on gluconeogenesis’s waste-clearing roles remain underexplored, potentially introducing subtle metabolic imbalances.

The hypothesis posited herein posits that metformin’s sustained inhibition of gluconeogenesis disrupts the Cori and Alanine cycles, leading to cumulative metabolite accumulation lactate, pyruvate, and ammonia that fosters low-grade inflammation through intricate molecular cascades. This may manifest as multisystem dysfunction, encompassing neuromuscular fatigue from ATP deficits, cognitive impairments via neuroinflammatory pathways, endothelial dysfunction accelerating atherosclerosis, hepatic stress from urea cycle overload, and immune dysregulation promoting inflammaging [2][3][16].

Contextualized within T2DM’s pathophysiology, where basal inflammation and oxidative burden are already heightened, this reevaluation is imperative. As T2DM prevalence escalates amid aging populations and sedentary lifestyles, understanding these hidden drivers could refine therapeutic paradigms, balancing glycemic benefits against potential inflammatory liabilities without undermining metformin’s established utility. This review synthesizes physiological foundations, mechanistic insights, and clinical implications to advocate for a nuanced perspective on metformin therapy.

## 2. Physiological Roles of Gluconeogenesis in Metabolic Homeostasis

Gluconeogenesis, the de novo synthesis of glucose from non-carbohydrate substrates, serves not only as a critical mechanism for maintaining blood glucose levels during fasting but also as an essential metabolic homeostasis pathway that integrates waste clearance, energy provisioning, and redox balance. This process is predominantly hepatic and renal, with the liver contributing approximately 80–90% of total gluconeogenic flux under fasting conditions, while the kidneys handle the remainder, particularly in prolonged starvation or acidosis [17]. At the core of this pathway are the Cori and Alanine cycles, which exemplify gluconeogenesis’s role in interorgan metabolite shuttling and detoxification. The Cori cycle facilitates the recycling of lactate produced during anaerobic glycolysis in peripheral tissues, such as skeletal muscle and erythrocytes [2][3].

Lactate dehydrogenase (LDH) in these tissues catalyzes the reversible conversion of pyruvate to lactate, coupled with NADH oxidation to NAD+, thereby sustaining glycolytic flux under oxygen-limited conditions. Lactate is then released into the bloodstream and transported to the liver or kidneys [18]. In hepatocytes, LDH regenerates pyruvate from lactate, which enters the mitochondrial matrix via the mitochondrial pyruvate carrier (MPC2), a transmembrane protein that ensures efficient pyruvate import while maintaining proton gradients [19]. Pyruvate carboxylase (PC), a biotin-dependent enzyme activated by acetyl-CoA, carboxylates pyruvate to oxaloacetate (OAA) using ATP and bicarbonate [20]. OAA is subsequently decarboxylated and phosphorylated by phosphoenolpyruvate carboxykinase (PEPCK) to form phosphoenolpyruvate (PEP), bypassing the irreversible pyruvate kinase step of glycolysis. PEP progresses through enolase, phosphoglycerate mutase, and phosphoglycerate kinase to 3-phosphoglycerate, then to fructose-1,6-bisphosphate via aldolase and triose phosphate isomerase reversals [21]. The pathway culminates with glucose-6-phosphatase (G6Pase) hydrolyzing glucose-6-phosphate to free glucose, which is exported via GLUT2 transporters. This cycle prevents lactic acidosis by clearing excess lactate, maintaining extracellular pH, and averting activation of pH-sensitive inflammatory sensors. Moreover, it supports energy homeostasis during stress, such as exercise, where lactate flux can increase tenfold, providing up to 40% of hepatic glucose output [1][2][3].

Beyond lactate clearance, the Cori cycle intersects with immune function through Warburg-like metabolic shifts. Activated immune cells, including macrophages and T-lymphocytes, exhibit enhanced aerobic glycolysis, producing lactate even in normoxic conditions to prioritize rapid ATP generation and biosynthetic intermediates for proliferation [22][23]. This Warburg effect mirrors the cycle’s peripheral phase, where lactate is shuttled to gluconeogenic organs, ensuring glucose availability for immune surveillance during infection or inflammation [24]. Efficient lactate recycling also controls reactive oxygen species (ROS) by modulating NADH/NAD+ ratios, preventing mitochondrial electron transport chain overload and subsequent superoxide formation [25].

Complementing the Cori cycle, the Alanine cycle addresses nitrogen disposal and carbon recycling, integrating with the urea cycle to avert ammonia toxicity. In skeletal muscle, alanine aminotransferase (ALT) transaminates pyruvate with glutamate, derived from branched-chain amino acid catabolism, to form alanine and α-ketoglutarate [26]. Alanine, a non-toxic nitrogen carrier, is released into circulation and taken up by hepatocytes via specific transporters [27]. Hepatic ALT reverses the reaction, regenerating pyruvate for gluconeogenesis and glutamate [28]. Glutamate dehydrogenase (GDH) then deaminates glutamate to α-ketoglutarate and ammonia in the mitochondria, with ammonia entering the urea cycle [29]. The principal enzymes involved in the Cori and Alanine cycles and their physiological functions are summarized in Table 1.

**Table:** Table 1. Key enzymes of the Cori and Alanine cycles and their homeostatic roles.

Cycle/Pathway	Key Enzymes	Normal Role	Molecular Links to Homeostasis
Cori Cycle	LDH, PC, PEPCK	Lactate recycling to glucose	Prevents HIF-1α–driven IL-6, supports Crabtree effect in immune cells
Alanine Cycle	ALT, GDH	Nitrogen to urea, carbon reuse	Avoids ammonia toxicity, integrates with Krebs cycle for ATP
Shared	MPC2, G6Pase	Metabolite shuttling	Controls ROS, pH; disruption links to sepsis-like states

This cycle begins with carbamoyl phosphate synthetase I (CPS1) condensing ammonia with bicarbonate and ATP to carbamoyl phosphate, which ornithine transcarbamoylase (OTC) transfers to ornithine, forming citrulline [30]. Citrulline condenses with aspartate via argininosuccinate synthetase (ASS1) to argininosuccinate, cleaved by argininosuccinate lyase (ASL) to arginine and fumarate. Arginase hydrolyzes arginine to urea and ornithine, completing the cycle [31]. The Alanine cycle thus disposes of nitrogenous waste, preventing hyperammonemia that could impair mitochondrial function or induce encephalopathy [32]. Carbon skeletons from alanine feed into gluconeogenesis, linking proteolysis with glucose production during fasting [33].

Shared molecular elements, such as MPC2 for pyruvate shuttling and CREB-CRTC2 transcriptional regulation of PEPCK and G6Pase, underscore the cycles’ interdependence. CREB, phosphorylated by PKA in response to glucagon or epinephrine, recruits CRTC2 to enhance gluconeogenic gene expression, ensuring adaptive responses to hormonal cues [34]. These pathways collectively maintain pH, ATP levels, and ROS homeostasis; disruptions could lead to metabolic acidosis, oxidative damage, or inefficient energy partitioning. In physiological contexts, gluconeogenesis supports brain and cardiac energy demands, where glucose is preferred, and renal acid-base balance via ammoniagenesis [35].

## 3. Molecular Mechanisms of Metformin-Induced Gluconeogenesis Inhibition

Metformin, a biguanide antidiabetic agent, exerts its primary therapeutic effect by suppressing hepatic gluconeogenesis, thereby reducing endogenous glucose production in type 2 diabetes mellitus (T2DM) [36].

This inhibition targets multiple molecular nodes within the gluconeogenic pathway, disrupting the Cori and Alanine cycles without directly affecting glycolysis or insulin secretion. At the cellular level, metformin’s action begins with its accumulation in hepatocytes via organic cation transporter 1 (OCT1), leading to mitochondrial perturbation [2][3]. The cornerstone mechanism involves inhibition of mitochondrial respiratory chain complex I (NADH:ubiquinone oxidoreductase), which impairs electron transfer from NADH to ubiquinone, reducing proton pumping and ATP synthesis [37]. This elevates the AMP/ATP ratio, activating AMP-activated protein kinase (AMPK) through phosphorylation at Thr172 by liver kinase B1 (LKB1) [38]. AMPK, a cellular energy sensor, phosphorylates downstream effectors to curtail energy-consuming processes. Specifically, AMPK targets protein kinase C, which phosphorylates CREB-binding protein (CBP) at Ser436 [39]. This modification disrupts the interaction between cAMP response element-binding protein (CREB) and CBP, preventing CREB-mediated transcription of gluconeogenic genes [40]. CREB, activated by glucagon-induced cAMP elevation and protein kinase A (PKA) phosphorylation, normally recruits CRTC2 (CREB-regulated transcription coactivator 2) to promoters of PEPCK and G6Pase, enhancing their expression [41]. Metformin-induced AMPK activation also promotes CRTC2 phosphorylation and nuclear exclusion, further repressing transcription [42].

Beyond transcriptional regulation, metformin modulates substrate-specific effects on gluconeogenesis. It suppresses lactate- and glycerol-dependent glucose production more profoundly than alanine-dependent flux, reflecting differential impacts on cycle intermediates [43]. In the Cori cycle, metformin reduces PC activity indirectly via AMPK-mediated inhibition of acetyl-CoA carboxylase (ACC), lowering acetyl-CoA levels that allosterically activate PC [2][44]. Additionally, metformin alters redox states by increasing NADH/NAD+ ratios through complex I blockade, impairing malate-aspartate shuttle function and limiting cytosolic OAA availability for PEPCK [45]. For the Alanine cycle, metformin’s effects are subtler; while it downregulates PEPCK and G6Pase, alanine transamination via ALT remains intact, allowing partial carbon recycling [3][46]. However, ammonia handling via GDH may be affected by metformin’s influence on mitochondrial energetics, potentially straining urea cycle integration [47]. A redox-dependent mechanism involves inhibition of mitochondrial glycerol-3-phosphate dehydrogenase (GPD2), which shuttles reducing equivalents from cytosolic NADH to the electron transport chain. Metformin’s suppression of GPD2 elevates cytosolic NADH, inhibiting lactate dehydrogenase and pyruvate entry into gluconeogenesis, while sparing alanine pathways that bypass this shuttle [48]. Furthermore, metformin impairs mitochondrial pyruvate carrier 2 (MPC2) function, restricting pyruvate import and exacerbating substrate accumulation [49].

This carrier, a heterodimer with MPC1, facilitates proton-coupled pyruvate transport; metformin’s mitochondrial effects may disrupt MPC2 conformation or expression, amplifying inhibition [50]. These mechanisms are context-dependent, with metformin concentrations (typically 1–5 mM in portal vein post-dose) achieving therapeutic effects without overt toxicity. In T2DM, where gluconeogenesis is upregulated due to insulin resistance and elevated glucagon, metformin’s actions restore balance by reducing fasting hyperglycemia. Additional pleiotropic effects include gut microbiota modulation, increasing short-chain fatty acid production that indirectly suppresses hepatic glucose output via AMPK-independent pathways. Overall, metformin’s multifaceted inhibition underscores its efficacy, though long-term implications on cycle dynamics warrant consideration [51].

## 4. Downstream Molecular Pathways Leading to Low-Grade Inflammation

Sustained disruption of gluconeogenesis by metformin, while beneficial for glycemic control, may inadvertently trigger downstream molecular pathways culminating in low-grade inflammation through metabolite accumulation. Lactate, pyruvate, and ammonia buildup from impaired Cori and Alanine cycles activates pro-inflammatory cascades, including hypoxia-inducible factor-1α (HIF-1α), nuclear factor-κB (NF-κB), and NLRP3 inflammasome, fostering a chronic inflammatory milieu in T2DM [52][53].

Lactate accumulation stabilizes HIF-1α by inhibiting prolyl hydroxylase domain enzymes (PHDs), which require oxygen and α-ketoglutarate for HIF-1α degradation [54]. Stabilized HIF-1α translocates to the nucleus, heterodimerizing with HIF-1β to transcribe genes like IL-6 and VEGF, promoting cytokine release and angiogenesis [55]. This pathway exacerbates inflammaging, where persistent low-level inflammation accelerates aging-related pathologies. Pyruvate excess elevates ROS via mitochondrial overload; impaired complex I function uncouples electron transport, generating superoxide from leaked electrons [56]. ROS activate NF-κB by phosphorylating IκB kinase (IKK), degrading IκBα and freeing NF-κB (p65/p50) for nuclear entry [57]. NF-κB induces TNF-α, IL-1β, and adhesion molecules, amplifying endothelial activation and leukocyte recruitment [58]. Ammonia from disrupted nitrogen disposal assembles NLRP3 inflammasome; elevated ammonia triggers lysosomal destabilization and cathepsin B release, priming NLRP3 [59]. ROS and potassium efflux further activate NLRP3, recruiting ASC and procaspase-1 to form the inflammasome complex [60]. Caspase-1 cleaves pro-IL-1β and pro-IL-18 to mature forms, secreted via gasdermin D pores, perpetuating inflammation [61]. Contextually, short-term AMPK activation by metformin may mitigate inflammation via NF-κB inhibition, but prolonged cycle disruption imposes “inflammatory debt”, particularly in mitochondrial-impaired states.

## 5. Multisystem Consequences of Disrupted Cycles

## 5.1. Musculoskeletal System

Disrupted Cori cycle function leads to lactate and pyruvate accumulation in skeletal muscle, impairing ATP production due to metformin’s inhibition of mitochondrial complex I [62]. This ATP deficit reduces myosin-actin cross-bridge cycling, resulting in muscle fatigue and weakness [63][64]. Elevated reactive oxygen species (ROS) from pyruvate excess trigger oxidative damage to myofibrillar proteins, promoting sarcolemmal senescence and myopathy [65]. Additionally, lactate-driven acidosis activates acid-sensing ion channels (ASICs), amplifying pain signaling via TRPV1 receptors, contributing to exercise intolerance and chronic muscle discomfort in T2DM patients [66].

## 5.2. Neurological System

Ammonia accumulation from impaired Alanine cycle nitrogen disposal disrupts astrocytic glutamate uptake, leading to excitotoxicity and neuronal hyperexcitability [67]. Elevated ammonia also induces astrocyte swelling by increasing glutamine synthesis, mimicking hepatic encephalopathy-like symptoms, such as cognitive fog and impaired attention [68]. ROS and IL-1β, driven by NLRP3 inflammasome activation, cross the blood-brain barrier, promoting microglial activation and synaptic dysfunction [69]. These effects impair neurotransmitter release, particularly acetylcholine and GABA, compromising cognitive processing and memory consolidation in susceptible T2DM subsets [70].

## 5.3. Cardiovascular System

Lactate excess stabilizes HIF-1α, upregulating vascular endothelial growth factor (VEGF) and adhesion molecules like VCAM-1, which enhance leukocyte adhesion and endothelial dysfunction [71]. NF-κB activation, triggered by ROS from pyruvate overload, induces endothelial apoptosis and reduces nitric oxide synthase (eNOS) activity, impairing vasodilation [72]. These changes promote atherosclerotic plaque formation and increase vascular stiffness, elevating risks of hypertension and myocardial infarction. Chronic low-grade inflammation further exacerbates oxidative stress, accelerating cardiovascular remodeling in T2DM [73].

## 5.4. Hepatic System

Ammonia buildup from disrupted Alanine cycle function stresses the urea cycle, overwhelming carbamoyl phosphate synthetase I (CPS1) and glutamate dehydrogenase (GDH) [74]. This leads to mitochondrial dysfunction, as ammonia inhibits Krebs cycle enzymes like isocitrate dehydrogenase, reducing ATP production [75]. Oxidative stress from ROS accumulation damages hepatocellular membranes, promoting lipid peroxidation and early fibrotic changes [76]. These alterations increase the risk of non-alcoholic fatty liver disease (NAFLD) progression to steatohepatitis, particularly in T2DM patients with pre-existing hepatic stress [77].

## 5.5. Immune System

Lactate accumulation modulates macrophage polarization, shifting M2 (anti-inflammatory) to M1 (pro-inflammatory) phenotypes via HIF-1α-mediated transcription of IL-6 and TNF-α [78]. This disrupts immune homeostasis, promoting inflammaging, a chronic inflammatory state linked to aging-related pathologies. Impaired Cori cycle function also limits glucose availability for activated leukocytes, compromising immune surveillance during infections [79]. NLRP3 inflammasome activation, driven by ammonia and ROS, amplifies IL-1β and IL-18 release, increasing susceptibility to sepsis-like systemic inflammatory responses in T2DM [80].

These multisystem effects highlight the far-reaching consequences of disrupted gluconeogenic cycles, extending beyond metabolic control to systemic dysfunction. The interplay of metabolite accumulation and inflammatory signaling underscores the need for a nuanced understanding of metformin’s long-term impact in T2DM management. The organ-specific molecular and functional consequences of disrupted Cori and Alanine cycles are summarized in Table 2.

**Table:** Table 2. System-specific molecular consequences of Cori and Alanine cycle disruption.

System	Key Disruption	Molecular Pathway	Potential Outcome
Musculoskeletal	Lactate, pyruvate accumulation	ROS-induced myofibrillar damage, ASIC activation	Muscle fatigue, myopathy, exercise intolerance
Neurological	Ammonia, ROS accumulation	NLRP3-driven IL-1β release, astrocyte swelling	Cognitive fog, memory impairment
Cardiovascular	Lactate excess, HIF-1α upregulation	NF-κB-mediated eNOS suppression	Atherosclerosis, vascular stiffness, hypertension
Hepatic	Ammonia overload, GDH stress	Mitochondrial dysfunction, lipid peroxidation	NAFLD progression, early fibrosis
Immune	Lactate-driven macrophage shift	HIF-1α-induced cytokine dysregulation	Inflammaging, sepsis susceptibility

## 6. Clinical Evidence and Observational Studies

In the musculoskeletal domain, studies report increased fatigue and myopathy in T2DM patients on long-term metformin therapy, particularly those with renal impairment [81]. Cohort studies have observed elevated plasma lactate levels (1.5–2.5 mmol/L above baseline) in approximately 15% of metformin users [82], correlating with reduced exercise capacity and muscle pain, likely due to ATP deficits and acidosis [83]. These symptoms are more pronounced in patients with mitochondrial dysfunction or coexisting myopathies [84].

Neurologically, observational studies indicate mixed outcomes. Some randomized controlled trials (RCTs) report a 35% reduction in dementia risk among metformin users, attributed to improved insulin signaling and reduced cerebral glucose hypometabolism [85]. However, subsets of patients, particularly those with prolonged use (>5 years), exhibit cognitive fog and impaired executive function, with biomarkers showing elevated IL-1β and ammonia levels in cerebrospinal fluid, suggesting neuroinflammatory contributions [86]. These effects are more prevalent in older adults or those with vitamin B12 deficiency, a known metformin side effect [87].

Cardiovascular studies highlight metformin’s dual effects. RCTs demonstrate a 10–15% reduction in myocardial infarction risk and improved endothelial reactivity, linked to short-term AMPK-mediated suppression of inflammatory cytokines like TNF-α and CRP [88]. Conversely, longitudinal cohorts report increased vascular inflammation markers (e.g., VCAM-1, E-selectin) in long-term users, particularly those with chronic kidney disease, suggesting a cumulative inflammatory burden from lactate-driven HIF-1α activation [89]. Hepatically, metformin is associated with rare but serious cases of lactic acidosis, with an incidence of 3–10 per 100,000 patient-years, predominantly in patients with renal or hepatic impairment [90]. Observational data link prolonged metformin use to elevated ammonia levels in 5–10% of T2DM patients with NAFLD, correlating with early fibrotic changes and urea cycle stress, as evidenced by increased serum AST/ALT ratios [91]. These findings suggest heightened hepatic vulnerability in predisposed individuals. In the immune system, clinical studies note altered immune responses in metformin-treated patients. Increased IL-6 and IL-1β levels in subsets with high lactate profiles indicate macrophage polarization toward pro-inflammatory states, potentially increasing sepsis risk during infections [92]. Conversely, short-term studies show reduced CRP and IL-6 in newly diagnosed T2DM patients, highlighting duration-dependent effects [93]. These findings suggest that while metformin offers anti-inflammatory benefits early in therapy, prolonged use may exacerbate inflammaging in certain contexts.

Overall, clinical evidence underscores the complex balance between metformin’s therapeutic benefits and its potential to induce multisystem complications through disrupted metabolic cycles, particularly in vulnerable populations.

## 7. Discussion

Gluconeogenesis, beyond its role in glucose production, serves as a critical metabolic scavenger, recycling lactate through the Cori cycle and nitrogenous compounds via the Alanine cycle to prevent acidosis, oxidative stress, and ammonia toxicity. The hypothesis posits that metformin’s inhibition of gluconeogenesis in type 2 diabetes mellitus (T2DM), achieved through mitochondrial complex I blockade, AMP/ATP ratio elevation, and AMPK-PKCι/λ-mediated repression of PEPCK and G6Pase, disrupts these cycles, leading to metabolite accumulation that drives low-grade inflammation and multisystem dysfunction. This “inflammatory debt” arises from lactate-induced HIF-1α stabilization upregulating IL-6 and VEGF, pyruvate-derived reactive oxygen species (ROS) activating NF-κB for TNF-α induction, and ammonia-triggered NLRP3 inflammasome assembly promoting IL-1β and IL-18 release. These pathways contribute to musculoskeletal fatigue from ATP deficits and ROS-mediated myopathy, neurological cognitive impairments via excitotoxicity and astrocyte swelling, cardiovascular endothelial dysfunction accelerating atherosclerosis through NF-κB/eNOS suppression, hepatic urea cycle overload risking fibrosis, and immune inflammaging from macrophage polarization shifts. Representative clinical observations and reported system-level outcomes associated with cycle disruption are summarized in Table 3.

**Table:** Table 3. Clinical observations associated with disruption of gluconeogenic cycles.

Domain	Key Observation	Notes/Implications
Musculoskeletal	Elevated lactate, fatigue in ~15% of users	Linked to ATP deficits; worsened in renal impairment
Neurological	Reduced dementia risk; cognitive fog in long-term use	IL-1β and ammonia rise in subsets; B12 deficiency risk
Cardiovascular	10–15% lower myocardial infarction risk; vascular inflammation	Short-term anti-inflammatory effects vs. long-term VCAM-1 rise
Hepatic	Lactic acidosis (3–10/100,000 patient-years), ammonia elevation	NAFLD progression risk in 5–10% of users
Immune	Pro-inflammatory shift in long-term users	Increased IL-6, IL-1β; higher sepsis susceptibility

Clinical evidence underscores this dual profile: short-term metformin use reduces cytokines like IL-6 and CRP through AMPK activation and gut microbiota modulation, improving cardiovascular outcomes, while long-term use in vulnerable populations (e.g., those with renal or hepatic impairment) correlates with lactic acidosis, cognitive fog, and elevated inflammatory markers. Key limitations include interindividual variability, driven by genetic factors such as OCT1 polymorphisms affecting metformin uptake or MPC2 variants altering pyruvate shuttling, and context-dependent effects influenced by comorbidities. These factors may exacerbate metabolite accumulation, amplifying inflammatory risks.

To mitigate these consequences, innovative strategies are proposed. Co-administration of N-acetylcysteine (NAC) could neutralize ROS, attenuating NF-κB and NLRP3 activation [94]. Pharmacological enhancement of mitochondrial pyruvate carrier (MPC2) activity via small-molecule agonists may restore pyruvate clearance, supporting Cori cycle function without compromising glycemic control [95]. Combining metformin with glucagon-like peptide-1 receptor agonists (GLP-1RAs), such as liraglutide, could provide neuroprotective and insulin-sensitizing benefits, counteracting cycle disruption [96]. Dietary interventions promoting short-chain fatty acid production may amplify metformin’s anti-inflammatory effects. Precision medicine, leveraging biomarker monitoring of lactate, IL-1β, and ammonia, could tailor dosing regimens to minimize inflammatory liabilities, particularly in patients with mitochondrial or renal dysfunction. These approaches validate the hypothesis by addressing the hidden costs of cycle disruption while preserving metformin’s cornerstone role in T2DM management, offering a framework for optimizing therapeutic outcomes.

## 8. Conclusion

Metformin’s inhibition of gluconeogenesis in T2DM disrupts the Cori and Alanine cycles, leading to lactate, pyruvate, and ammonia accumulation that drives low-grade inflammation via HIF-1α, NF-κB, and NLRP3 pathways. This contributes to multisystem dysfunction, including muscle fatigue, cognitive impairment, atherosclerosis, hepatic stress, and immune dysregulation. While short-term AMPK-mediated cytokine reduction and cardiovascular benefits are evident, prolonged use may incur an “inflammatory debt”, particularly in patients with renal or mitochondrial impairments. Clinical evidence underscores this balance, highlighting metformin’s indispensable role in glycemic control alongside potential risks. Precision medicine, integrating biomarker monitoring and adjunct therapies like antioxidants or GLP-1RAs, could mitigate these liabilities, optimizing long-term outcomes. This nuanced perspective calls for further research to refine metformin’s application, ensuring its therapeutic benefits are maximized while addressing hidden inflammatory costs in T2DM management.

## Statements and Declarations

Funding information: The authors received no financial support for the research and publication of this article.

Competing interest declaration: The authors declare that there are no conflicts of interest.

Author contribution: Maher Monir Akl: conceived and developed the research idea, performed the chemical synthesis, data analysis, and drafted the manuscript. Amr Ahmed: supervised the clinical aspects, contributed clinical expertise and critical revision of the manuscript.
